# Scale dependence of structure-function relationship in the emphysematous mouse lung

**DOI:** 10.3389/fphys.2015.00146

**Published:** 2015-05-12

**Authors:** Susumu Sato, Erzsébet Bartolák-Suki, Harikrishnan Parameswaran, Hiroshi Hamakawa, Béla Suki

**Affiliations:** ^1^Department of Biomedical Engineering, Boston UniversityBoston, MA, USA; ^2^Department of Respiratory Medicine, Kyoto University HospitalKyoto, Japan

**Keywords:** heterogeneity, airspace diameter, lung, compliance, inflammation

## Abstract

The purpose of this study was to determine how the initial distribution of elastase in mouse lungs determines the time course of tissue destruction and how structural heterogeneity at different spatial scales influences lung function. We evaluated lung function and alveolar structure in normal and emphysematous C57BL/6 mice at 2 and 21 days following orotracheal treatment with porcine pancreatic elastase (PPE). Initial distribution of elastase 1 h after treatment was assessed using red fluorescently labeled PPE (*f*-PPE) by laser scanning confocal microscopy. From measured input impedance of the respiratory system, the global lung compliance, and the variability of regional compliance were obtained. Lungs were fixed and equivalent airspace diameters were measured in four lobes of the right lung and three regions of the left lung. At day 2 and day 21, the mean airspace diameter of each region was significantly enlarged which was accompanied by an increased inter-regional heterogeneity. The deposition of *f*-PPE on day 0 was much more heterogeneous than the inter-regional diameters at both day 2 and day 21 and, at day 21, this reached statistical significance (*p* < 0.05). Microscale heterogeneity characterized by the overall variability of airspace diameters correlated significantly better with compliance than macroscale or inter-regional heterogeneity. Furthermore, while the spatial distribution of the inflammatory response does not seem to follow that of the elastase deposition, it correlates with the strongest regional determinant of lung function. These results may help interpret lung function decline in terms of structural deterioration in human patients with emphysema.

## Introduction

Mouse models are useful for investigating the mechanisms of disease pathogenesis or progression. Emphysema is in particular a human disease that has been studied with the help of mouse models (Fisk and Kuhn, 1976; Gardi et al., 1992; De Santi et al., 1995; O'donnell et al., 1999; Lucattelli et al., 2003; Shiomi et al., 2003; Cantor et al., 2005; Foronjy et al., 2005; Yao et al., 2010; Hamakawa et al., 2011). The cigarette smoke-induced effects of enzymes in the lung are often mimicked by treating mice with elastase (Lucattelli et al., 2003; Ito et al., 2005; Hantos et al., 2008; Yao et al., 2010; Hamakawa et al., 2011). While this model has obvious limitations, it is useful to investigate the time course of structural changes in the lung tissue due to the fast progression of emphysema.

Following treatment of the lung with elastase, the parenchymal structure is gradually destroyed. The first sign of change is the appearance of structural heterogeneity (Parameswaran et al., 2006, 2009) which has been attributed to alveolar wall rupture (Kononov et al., 2001). However, it is conceivable that the distribution of local structural changes due to elastase instillation also depends on the preferential deposition of elastase and not only the actual mechanism of airspace enlargement. Therefore, understanding how the initial distribution of elastase in the lung influences the time course of the development of regional heterogeneity of tissue destruction could help better understand the mechanism of airspace enlargement itself. It is possible that mechanical failure causes small scale heterogeneity at the level of tens of alveoli whereas the initial distribution of elastase contributes to large scale heterogeneity such as inter-lobar variations in structure. However, it is not known which of these processes dominate the time course of the overall structural deterioration of the lung.

Computational modeling suggests that the topographical distribution of tissue destruction influences function (Parameswaran et al., 2011). Indeed, in patients with mild emphysema, the severity of structural abnormalities showed correlations with the degree of hypoxemia and ventilation-perfusion mismatch, but the relationship disappeared during exercise (Barbera et al., 1991). Also, macroscale structural pattern such as centrilobular or panacinar emphysema has important consequences on the mechanical properties of the lung in human emphysema (Saetta et al., 1994). Nevertheless, it is not known how structural heterogeneity at different spatial scales influences lung function.

The purpose of this study was to investigate the length scale dependence of structure-function relationship in emphysematous mouse lungs following elastase treatment. Specifically, we aimed at determining how heterogeneity at different length scales influences lung function and whether such relations change during the progression of emphysema. To this end, we measured the initial distribution of fluorescent elastase immediately following orotracheal administration and compared its spatial distribution with the heterogeneity of tissue structure at large and small scales in the mouse lung at two time points following treatment. This comprehensive analysis of lung structure also allowed us to investigate the spatial scales of structural heterogeneity that best correlates with lung function.

## Methods

### Animal preparation

Procedures were approved by the Animal Care and Use Committee of Boston University. Four groups of C57BL/6J mice (Charles River Laboratories, Boston, MA) were used. The first group received no treatment and served as the control group (*n* = 6). The rest of the mice were initially anesthetized with isoflurane on the day of treatment. The second group (*n* = 5) was treated oropharyngeally with porcine pancreatic elastase (PPE; Elastin Products Company, Owensville, MO) using a dose of 7.5 IU dissolved in 100 μl phosphate buffered saline. The PPE was red fluorescently labeled (*f*-PPE) using a Dylight® labeling kit (Pierce, Rockford, IL) as previously described (Jesudason et al., 2010). Experiments were carried out 1 h after the treatment to determine the initial spatial distribution of elastase deposition throughout the lung. The third group (*n* = 6) and the fourth group (*n* = 6) received oropharyngeal treatment of unlabeled PPE (7.5 IU) and experiments were carried out 2 and 21 days after the treatment, respectively.

On the day of the experiments, mice were anesthetized with intraperitoneal injection of pentobarbital sodium (70 mg/kg), tracheostomized and then cannulated with an 18-guage steel needle in the supine position. The cannula was connected to a computer-controlled small animal ventilator (flexiVent, SCIREQ, Montreal, Quebec, Canada) and the animals received ventilation with a tidal volume of 8 ml/kg at a frequency of 240 breaths/min.

### Respiratory mechanics

**Figure 1:**
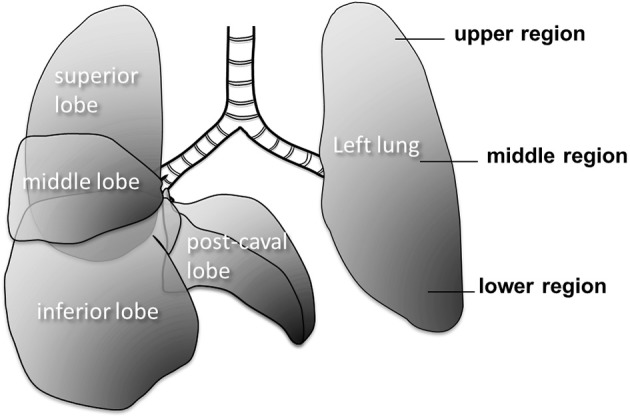
**Large scale structure of the mouse lung**. The right lung has 4 lobes and the left lung has one lobe. The right lung consists of superior, middle, inferior and post-caval lobes.

Airway opening pressure and flow delivered to the mice were sampled by flexiVent system while delivering forced oscillations according to the optimal ventilation waveform approach (Lutchen et al., 1993) at 0 and 3 cmH_2_O positive end-expiratory pressure (PEEP). Input impedance of the respiratory system (Zrs) was then computed from the Fourier transforms of pressure and flow. To standardize volume history, each measurement was preceded by two inflations to 25 cmH_2_0 airway pressure. The Zrs spectra were fit with a model composed of Newtonian resistance (R), airway inertance (I_aw_) and the constant phase tissue impedance (Hantos et al., 1992) connected in series to obtain respiratory tissue resistance (G) and elastance (H) parameters. The Zrs was also fit with a more complex inverse model that included a parallel set of pathways with distributed elastance (Ito et al., 2004). The model structure is shown in Figure 1 of the Complementary data. The model assumes that the tissue component in each pathway has the same hysteresivity (Fredberg and Stamenovic, 1989) defined as G/H. Furthermore, the regional tissue compliance C=1/H, that is the compliance in a given pathway, is distributed between a minimum (C_min_) and a maximum (C_max_) value in a hyperbolic manner. The input impedance of this model can be analytically calculated and the formula for impedance as a function of model parameters can be fit to measured impedance data (Ito et al., 2004). This analysis then provides estimates of G, C_min_, C_max_, R, and I_aw_ and the mean and standard deviation (SD) of regional compliance (SD of C) can be calculated from C_min_, C_max_ and the hyperbolic nature of the distribution of C. In this study, we limited the analysis to the mean C and the SD of C.

### Morphometry

Quantitative morphometry was carried out in seven distinct regions of the lung of each mouse (Figure 1). Fluorescent images (see below) were taken from the four lobes of the right lung as well as three regions (upper, middle, and lower) of the left lung. Since the left lung is much larger than the lobes of the right lung, the three regions of the left lung were nearly as big as the lobes of the right lung. These seven regions will be collectively called as macro regions.

The lungs that received *f*-PPE were gently perfused with 2 ml of PBS via the right ventricle and were isolated. All four lobes of the right lung and three regions of left lung were dissected perpendicular to the direction of body axis. The cut surface was then placed in the dish and saline buffer was added. Using a laser scanning confocal microscope (Olympus FLUOVIEW®FV-1000), alveoli were visualized at a depth of at least 50 μ so as to minimize the effect of the uneven cut surface. At least three images were randomly selected in each lobe and region and imaged to simultaneously map alveolar structure and the distribution of *f*-PPE. Tissue auto fluorescence was excited by a 488 nm laser and emission collected between 500 and 600 nm (Ch1). The *f*-PPE was excited by a 633 nm laser and emission collected between 601 and 665 nm. To assess the distribution of instilled *f*-PPE, first a mask image of the lung field was created from the auto fluorescent image then the total signal intensity of f-PPE was measured over the area of the mask.

**Figure 2:**
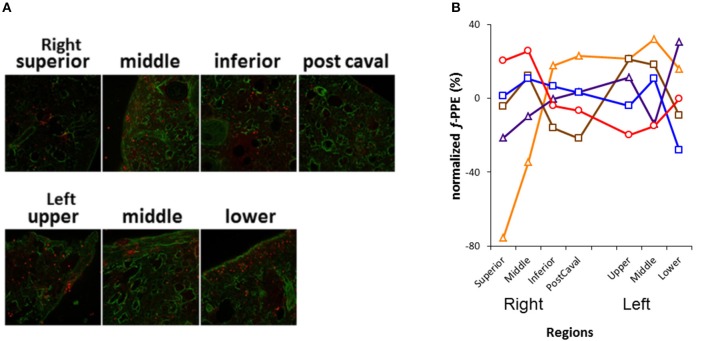
**Analysis of the distribution of fluorescently labeled porcine pancreatic elastase (f-PPE)**. **(A)** Representative merged confocal images in each lobe of the right lung and the three regions of the left lung are displayed. The green represents tissue auto fluorescence whereas the red shows f-PPE. **(B)** Inter-lobe variability of f-PPE. Each line corresponds to an individual mouse. Each plot is normalized by the average intensity of f-PPE in the mouse and displayed as % deviation from the average. A value >0% means that more f-PPE was found in a given lobe or region, while a value <0% means less amount of f-PPE than the average value in that mouse.

In the remaining groups, the lungs were perfused, isolated and then fixed in 10% formalin at 30 cmH_2_O airway pressure. Randomly selected regions were imaged. Tissue autofluorescence was used to characterize structure. The images were automatically segmented and the area of the airspaces and the equivalent diameter (D*_eq_*) of airspaces were measured. The minimum and average number of airspaces per region was 135 and 423, respectively. Figure 2 in the Complementary data summarizes the image processing and computations. The D_eq_ is defined as the diameter of a circle with the same area as the selected airspace. The mean of the equivalent diameters (D) and the area weighted mean equivalent diameter (D_2_) (Parameswaran et al., 2006) were also calculated. The D_2_ is sensitive to both the increase in size and heterogeneity of airspaces. All image analyses were conducted by custom program running on MATLAB (Mathworks, Natick, MA).

We also calculated the mean diameter for all seven macro regions (i.e., the four lobes of the right lung and the three regions of the left lung) and the corresponding mean diameters will be denoted by D_reg_. The D_reg_ thus represents average airspace diameter in one of the seven regions of the mouse lung and the SD of the 7 D*_reg_* values in a given animal can be considered as the large scale or “macroscale” variability of tissue destruction. In contrast, small scale or “microscale” heterogeneity of destruction was characterized by the SD of D and D_2_ of each region in each animal. In addition, to compare microscale heterogeneity with macroscale heterogeneity, we computed the coefficient of variation of D_eq_ and D_reg_, respectively.

### Immunohistochemistry

The abundance of several inflammatory cell types was evaluated at 2 and 21 days after PPE injury from the superior and inferior lobes (Figure 1). The macrophages and lymphocytes were visualized by an antibody complex rat anti-CD16+CD32 (Abcam Inc. Cambridge, MA) detecting the conformational epitope formed by CD16 Fc gamma II and CD32 Fc gamma III receptors. The activated T-cells, B-cells, and monocytes were visualized by an antibody recognizing the activated leukocyte cell adhesion molecule (ALCAM/CD166, Santa Cruz Biotechnology Inc, Dallas, Tx). Formalin (10%, neutral buffered) fixed, paraffin-embedded sections were deparaffinized in xylene and rehydrated in decreasing alcohol series. Endogenous peroxidase activity was quenched by 1% H_2_O_2_ and sections were washed in 10 mM sodium phosphate buffer, 150 mM NaCl (PBS), pH 7.5. A blocking step was performed with horse serum and sections were incubated for 1 h with one of the primary antibodies. Rat or rabbit IgG (20 ng/ml) as well as omitting the primary or secondary antibodies were used as technical controls. After PBS washes, the rat or rabbit HRP conjugated secondary antibodies (Vector Lab, Burlingame CA) were applied for 1 h. Sections were washed in PBS and incubated for 30 min in VECTASTAIN ABC reagent (Vector Lab). Enzyme substrates (Vector Lab) were applied until the right colors developed: DAB (brown) for CD16/32, and Vector SG (blue/gray) for ALCAM. After this step, counter staining (Nuclear Fast Red for ALCAM and Methyl Green for CD16/32, Vector Lab) and dehydration-clearing-mounting was applied. All conditions were processed simultaneously for each antibody (*n* = 30/condition). Images were captured by a Nikon Eclipse 50i microscope and SPOT camera (Micro Video Instruments, Avon, MA) and histological evaluation was performed.

### Statistical analysis

All data are presented as mean (SD). Different groups were tested with 1- or 2-Way ANOVA and paired or unpaired *t*-test using a statistical package (PASW Statistics 18.0, SPSS, Chicago, IL). Multivariate regression was used to identify the most relevant structural contributions to function. A significant difference was defined as *p* < 0.05.

## Results

**Figure 3:**
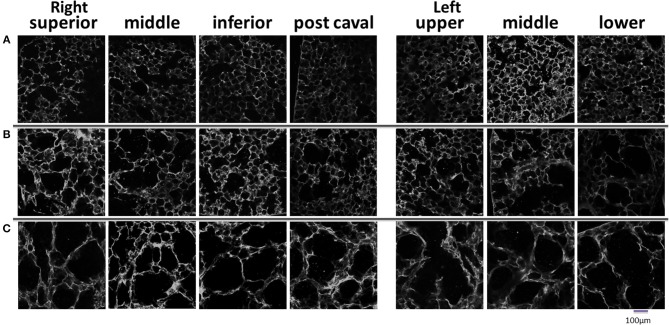
**Representative auto fluorescent confocal images of lung parenchyma in each lobe or region studied**. Normal mice **(A)**, day 2 mice **(B)**, and day 21 mice **(C)**.

**Figure 4:**
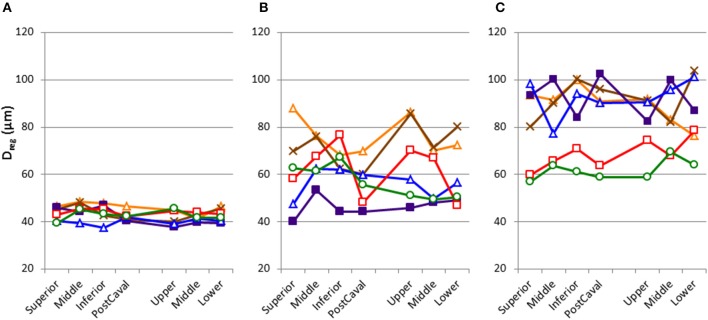
**Inter-regional variability of structure in each mouse as characterized by the equivalent diameters averaged over each region (D_reg_) with the regions defined in Figure 1**. Each line corresponds to an individual mouse. Normal mice **(A)** show homogeneous distribution. In contrast, elastase-treated mice **(B,C)** show increased mean and variability of D_reg_. Notice also that in the treated mice, the pattern was not uniform among individuals. Day 2 mice **(B)** and Day 21 mice **(C)** also shows differences.

Figure 2A shows a series of images demonstrating the spatial distribution of *f*-PPE whereas Figure 2B shows the variation of the intensity in the macro regions around the mean in each animal studied 1 h after treatment. There is significant inter-animal variation without any apparent pattern regarding the deposition of *f*-PPE in the different regions of the lung. Tissue structure in the various macro regions as defined in Figure 1 is exemplified in Figure 3 for the normal mice as well as for mice at days 2 and 21 days after treatment. The corresponding mean regional diameter (D_reg_) is shown in Figure 4. In the normal lung, D_reg_ is around 45 μm displaying little heterogeneity among the regions. Note that the normal lung also characterizes the tissue structure on day 0 just before the administration of *f*-PPE. In contrast, at day 2 and 21, the mean D_reg_ is around 60–80 μm, respectively, with a significant increase in inter-regional heterogeneity. It is noteworthy that there is no clear dependence of tissue destruction on location.

**Figure 5:**
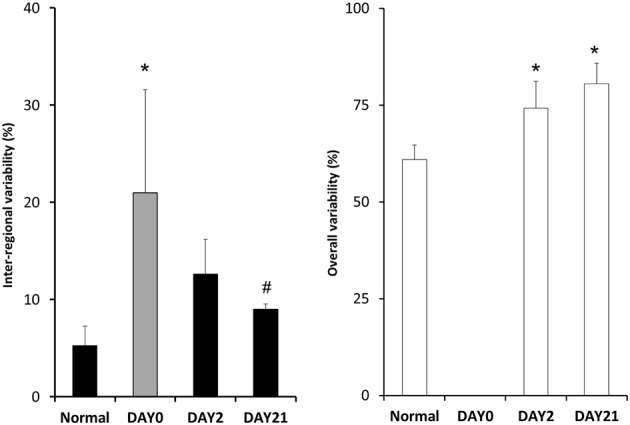
**The coefficients of variation of various structural indexes**. The gray bar shows the coefficient of variation of the mean *f*-PPE intensity for each region. The filled black bars represent the coefficient of variation of the mean equivalent diameter for each region (D_reg_) whereas the white bars correspond to the coefficient of variation of all equivalent diameters (D_eq_). Note that no data are presented for the overall variability on day 0 because it is expected to be similar to the normal case since f-PPE likely has no significant effect in one hour. ^*^: *p* < 0.05 vs. Normal; ^#^: *p* < 0.05 vs. Day0 (*f*-PPE).

The inter-regional variability of *f*-PPE deposition is compared with the variability of inter-regional mean diameter in Figure 5 using the coefficient of variation of the mean regional *f*-PPE intensity and D_reg_, respectively, which represent macroscale heterogeneity. It can be seen that the deposition of *f*-PPE on day 0 is much more heterogeneous than the inter-regional diameters at both day 2 and day 21 and, at day 21, this reached statistical significance (*p* < 0.05). Figure 5 also shows the coefficient of variation of all D_eq_, which represent microscale heterogeneity. In contrast to macroscale heterogeneity, the microscale variability steadily and significantly increases over time from 60% in normal mice to 80% at day 21 (*p* < 0.05).

Table 1 summarizes the average respiratory mechanical parameters in normal mice and mice 2 or 21 days after treatment with PPE. Except for R, the treatment had a significant effect on C, SD of C, G already at day 2 whereas at day 21 all parameters including I_aw_ were different from those in normal mice. Additionally, at day 21, most parameters are different from those at day 2 suggesting progressive worsening of function.

**Table 1:** **Mechanical properties of respiratory system**.

**Treat-ment**	**CP model**				**HTE model**
	**G (cmH_2_O/ml)**	**C (ml/cmH_2_O)**	**R (cmH_2_O/ml/s)**	**Iaw (cmH_2_O/ml/s)**	**SD of C (ml/cmH_2_O)**
Normal	4.99	0.034	0.238	0.00021	0.019
	(0.25)	(0.0014)	(0.030)	(4.15 × 10^−5^)	(0.0021)
Day 2	4.23^**^	0.044	0.232	0.00025	0.025^*^
	(0.48)	(0.0080^*^)	(0.035)	(4.21 × 10^−5^)	(0.0057)
Day 21	3.06^**^^#^	0.081^**^^#^	0.208	0.00031^*^	0.040^**^^#^
	(0.42)	(0.0123^**^)	(0.033)	(6.36 × 10^−5^)	(0.0028)

CP, constant phase model; HTE, heterogeneous tissue elastance model;

* p < 0.05 vs. Normal,

** p < 0.01 vs. Normal, and

# *p < 0.01 vs. Day 2. Data are shown as mean (SD)*.

**Figure 6:**
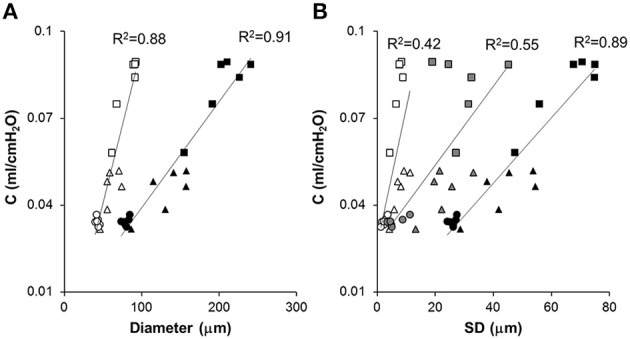
**Correlation between respiratory system compliance and structural parameters at different length scales**. Each symbol represents a mouse from the Normal (circle), Day 2 (triangle) or Day 21 (square) groups. **(A)** Respiratory system compliance (C) is correlated with the mean of all diameters (open symbols) and the area weighted mean of all diameters (black symbols). **(B)** The SD of D_reg_ (open symbols), overall SD of all equivalent diameters (black), and inter-regional SD of D_2_ (gray) are correlated with C The solid lines show separate linear regressions through the combined data of all mice.

**Figure 7:**
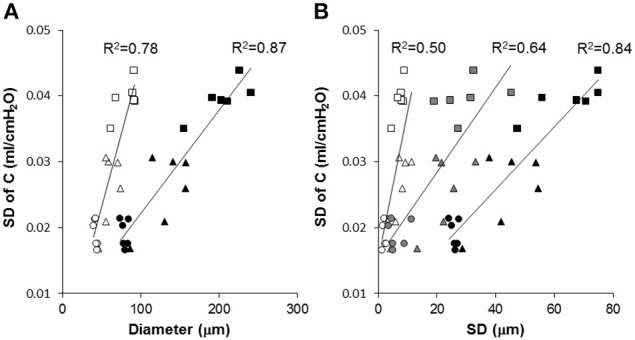
**Correlation between model-based estimate of functional heterogeneity, the SD of C, and structural parameters at different length scales**. Each symbol represents a mouse from the Normal (circle), Day 2 (triangle) or Day 21 (square) groups. **(A)** SD of C is correlated with the mean of all diameters (open symbols) and the area weighted mean of all diameters (black symbols). **(B)** The SD of D_reg_ (open symbols), overall SD of all equivalent diameters (black), and inter-regional SD of D_2_ (gray) are correlated with the SD of C. The solid lines show separate linear regressions through the combined data of all mice.

The compliance C and its SD are correlated with various structural descriptors in Figures 6, 7, respectively. It can be seen that for both cases, the overall D or D_2_ and the SD of inter-regional D_2_ correlate best with function (C or SD of C). In order to assess whether the correlations are dominated by a given region (lobes of the right lung or regions of the left lung), we carried out a multivariate regression analysis between C and the D or D_2_ of all regions (Table 2). Interestingly, this analysis consistently showed that the inferior lobe of the right lung and the lowest region of the left lung had the largest contribution to C. A similar analysis using SD of C resulted in slightly weaker structure-function correlations with the strongest determinant of the correlations due to the inferior lobe of the right lung and the middle region of the left lung (Table 3).

**Table 2:** **Multivariate regression analysis for respiratory compliance with regional equivalent diameter (D_reg_) or area weighted diameter (D_2_)**.

	**Model I (D_reg_)**		**Model II (D_2_)**		**Model II by stepwise**	
	**β**	**p**	**β**	**p**	**β**	**p**
R^2^	0.92^*^		0.99^*^		0.98^*^	
**INDEPENDENT VARIABLES**						
Right					-	
Superior	−0.11	NS	1.39	NS		
Middle	−0.17	NS	−2.66	0.024	-	
Inferior	2.24	0.049	6.06	<0.001	0.44	0.002
PostCaval	0.009	NS	−1.95	NS	-	
Left Lobe					-	
Upper	−1.50	NS	−0.39	0.006	-	
Middle	3.71	0.004	0.36	0.01		
Lower	1.47	NS	0.81	<0.001	0.56	<0.001

* *p < 0.01, model I; multiple regression with D_reg_ of each lobe. model II; multiple regression with D_2_ of each lobe*.

**Table 3:** **Multivariate regression analysis for regional compliance (SD of C) with regional equivalent diameter (D_reg_) or area weighted diameter (D_2_)**.

	**Model I (D_reg_)**		**Model I by stepwise**		**Model II (D_2_) by stepwise**	
	**β**	**p**	**β**	**p**	**β**	**p**
R^2^	0.87^*^		0.84^*^		0.89^*^	
**INDEPENDENT VARIABLES**						
Right					–	
Superior	−0.17	NS				
Middle	−0.12	NS			-	
Inferior	0.66	0.041	0.52	0.02	0.53	0.014
PostCaval	−0.49	NS			-	
Left Lobe					-	
Upper	−0.50	NS			-	
Middle	0.70	NS	0.43	0.048	0.43	0.04
Lower	0.62	NS				

* *p < 0.01, model I; multiple regression with D_reg_ of each lobe. model II; multiple regression with D_2_ of each lobe*.

**Figure 8:**
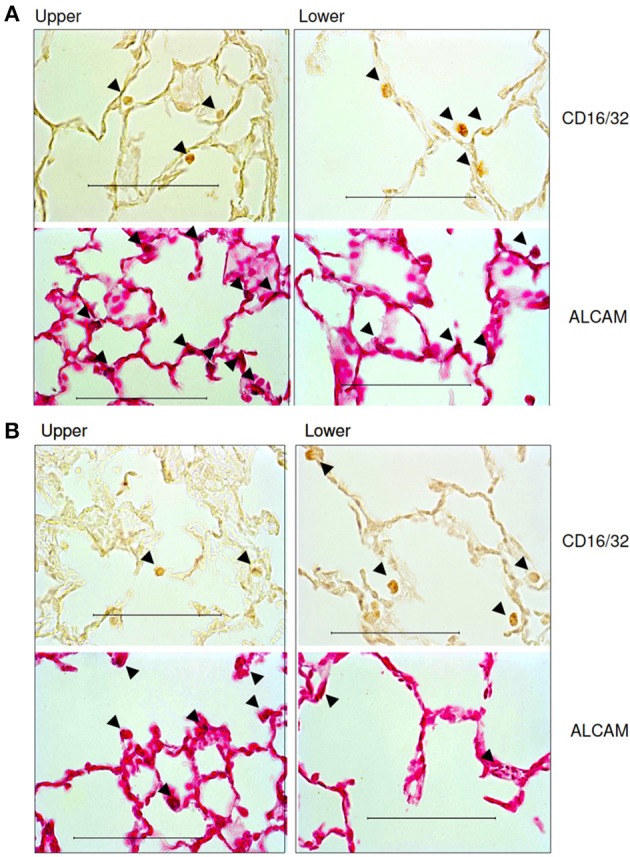
**Representative immunohistochemical images of the distribution of inflammatory cells in the upper and lower regions of the lung**. **(A)** Images at day 2 and **(B)** images at day 21. Semi-quantitative histological analysis revealed that the whole lung was infiltrated by inflammatory cells after PPE treatment and that the lower region showed a stronger inflammatory response.

Finally, using immunohistochemistry, we also evaluated the regional distribution of several inflammatory cells at day 2 and 21 (Figure 8). Table 4 summarizes the results. More macrophages and lymphocytes were seen in the inferior than the superior lobe at both time points. In contrast, more activated T-cells, B-cells and monocytes could be found in the superior lobe at both time points.

**Table 4:** **Semi-quantitative analysis of the spatial distribution inflammatory cells**.

	**Day 2**		**Day 21**	
	**CD16/32**	**ALCAM**	**CD16/32**	**ALCAM**
Superior Lobe	+++	++++++++	+++	+++++++
Inferior Lobe	++++	+++++	++++++	++

## Discussion

There are several studies that reported on the macroscale distribution of emphysematous lesions in human COPD patients. For example, upper lung predominant emphysema is quite common in cigarette smoke induced emphysema (Mohamed Hoesein et al., 2012), whereas lower predominance is more common in alpha1 antitrypsin deficiency patients (Bakker et al., 2008). A recent study also showed that there are some patients with a homogeneous pattern of distribution of emphysema and these patients appear to have a rapid decline in lung function (Tanabe et al., 2012). Small scale heterogeneity of tissue destruction pattern also has important consequences on the mechanical property of emphysema (Hamakawa et al., 2011). Furthermore, it has been shown in elastase treated rats that the relative area of low attenuation on CT images correlates with microscopic structural indexes such as the mean inter-wall distance which in turn correlates with dynamic lung compliance (Onclinx et al., 2006). Thus, while it appears that both macroscale and microscale heterogeneity of structure can influence function, it remains unclear how heterogeneity at various length scales in a given lung determines lung function.

The present study was designed to better understand how the initial spatial distribution of elastase-induced injury determines the evolution of structural destruction in the lung and how the heterogeneity of structure at various length scales influences function. Our main findings suggest that (1) The initial distribution of elastase is highly heterogeneous; (2) Indexes that are sensitive to heterogeneity (SD of all airspace sizes and D_2_) correlate best with overall lung compliance; (3) Inter-regional variability of mean airspace enlargement became more homogeneous as emphysema progressed and showed less correlation with function; (4) The inferior lobe showed the highest number of inflammatory cells and its structural destruction had the strongest effect on lung function characterized by the compliance and its SD.

Our results thus further advance the understanding of how structure determines function and how it occurs at various spatial length scales. Specifically, we found that in our elastase-induced mouse model of emphysema, large scale heterogeneity at the level of lobes has less influence on function than small scale heterogeneity. This may be a result of the averaging process going from small to large scales. For example, the D_2_ computed from all airspace diameters reached an R^2^ value of 0.91 (Figure 6A) much higher than the 0.73 found by Onclinx et al. between dynamic compliance and mean perimeter per unit area (Onclinx et al., 2006). Similarly, while static compliance was different between emphysema patients with centrilobular and panacinar pathology, neither the destructive index not the mean linear intercept showed any histological difference between these groups (Saetta et al., 1994). Interestingly, however, the coefficient of variation of alveolar wall distance steadily decreased as more of the lung destruction was panacinar-like (Saetta et al., 1994). The reason for the strong relation between lung function and D_2_ is that the latter incorporates the first three moments of the distribution of equivalent diameters and hence it is highly sensitive to both the overall increase in diameter and enhanced heterogeneity of structure (Parameswaran et al., 2006). We also evaluated how the model-based functional heterogeneity, SD of C, correlates with structural features (Figure 7). Again, the microscale heterogeneity characterized by D_2_ correlated best with SD of C reaching an R^2^ value of 0.87. This is a surprisingly high value given that SD of C is likely influenced by both compliance and airway heterogeneities whereas the model only takes into account compliance heterogeneities.

The airspace structure in emphysema has also been analyzed by Mishima et al. (1999) as a fractal structure which revealed that distribution of low attenuation areas follows a power law and the exponent of the power law is highly sensitive to the development of early emphysema in human patients. The exponent of a power law distribution is a single number that characterizes the self-similar nature of the tail of the distribution and hence it is sensitive to heterogeneities on all scales (Suki, 2002). Indeed, the fractal nature of the tissue structure is maintained at the level of alveoli (Sato et al., 2007). In other words, the fractal property already takes into account the multi-scale heterogeneity of enlarged airspace sizes which we find in this study to correlate strongly with lung function. An important practical implication is that indexes obtained at the macroscale can reflect heterogeneity at the microscale due to the self-similar nature of the structure and hence such indexes obtained from CT, microCT or MRI imaging should be good indicators of functional deterioration. Indeed, the helium diffusivity derived from MRI images showed a very strong correlation with the D_2_ of the underlying heterogeneous structure (Jacob et al., 2008). The increased heterogeneity in emphysema should strengthen the relation between structure and function with consequences such as ventilation heterogeneity (Emami et al., 2008) and particle deposition heterogeneity (Oakes et al., 2014). For example, microCT derived parameters do correlate with D_2_ in elastase-induced mouse model of emphysema (Artaechevarria et al., 2011). With regard to the mechanism that increases heterogeneity, Mishima et al. (1999) also showed that progression is consistent with the coalescence of small clusters of low attenuation areas which can be accounted for by mechanical forces rupturing alveolar walls. Thus, failure mechanics-induced structural destruction plays an important role in the decline of lung function during the progression of emphysema (Suki et al., 2003).

Our results also provide insight into the role of the initial distribution of elastase in the development of structural heterogeneity. The mouse right lung has four lobes and the left lung has one lobe (Figure 1). Elastase was given in a slanted body position. Therefore, if gravity was responsible for the flow of elastase solution down the airways, the lower regions of the lung (e.g., inferior lobe, post-caval lobe and lower region of the left lung) should have received more elastase causing lesions to preferentially develop in those regions. In fact, Figure 2B demonstrates that this was not the case: there was not substantially more elastase in the gravitationally preferred regions of the left lung than elsewhere. Thus, in accord with the liquid plug flow studies (Cassidy et al., 2001), the elastase was likely driven by airflow. Although the airflow-driven liquid should be distributed more homogeneously (Cassidy et al., 2001), our confocal images showed strong heterogeneity at a much smaller scale than the small airways in microfocal x-ray images. Nevertheless, Figure 2 appears to suggest that the superior lobe may have received less elastase than other regions although the deposition of elastase was highly heterogeneous. One possible reason for this finding is that immediately following the orotracheal instillation of elastase, the chest of the mouse was gently massaged that could help more uniformly distribute the elastase into all regions. In contrast, the regression analysis unequivocally showed that structural destruction in the inferior lobe and the lower region of the left lung was the most important determinant of C (Table 2) as well as the SD of C (Table 3). This is also supported by the increased number of macrophages and B lymphocytes seen in the inferior than the superior lobes at both time points (Table 4). It seems difficult to reconcile the discrepancy between the initial distribution of elastase and the fact that function seems to be determined by the gravitationally preferred regions. It is possible that minor differences in the initial distribution together with other not measured factors such as local blood flow, local mechanical stresses, mechanotransduction or locally existing minor inflammation may have attracted more inflammatory cells to release more enzymes that triggered structural destruction slightly more in the inferior and lower regions.

Macroscale heterogeneity of structure which was much smaller than overall microscale heterogeneity, decreased with time (Figure 5). In sharp contrast, both functional (Table 1) and microscale heterogeneity (Figure 5) kept increasing with the progression of emphysema. Consequently, microscale heterogeneity had a significantly stronger contribution to functional heterogeneity. Thus, despite the highly heterogeneous initial distribution of exogenous elastase, further proteolytic injury and mechanical failure will not necessarily localize to the initial site of elastase injury and eventually tissue destruction develops throughout the lung leading to a decrease in macroscale heterogeneity over time. This whole organ response is likely due to the development of inflammation throughout the lung followed by other mechanisms such as local apoptosis (Demedts et al., 2006), release of enzymes (Churg and Wright, 2005) and eventual rupture of septal walls (Kononov et al., 2001). Indeed, even the superior lobe that may have received less elastase (Figure 2B), exhibited a strong activation of inflammation judged by ALCAM (Table 4).

Before concluding, we note that it is customary to standardize mechanical measurements by inflating the lung to between 25 and 35 cmH_2_O once or twice. It may be argued that such a maneuver may lead to septal wall failure and hence an artifact of the measurement protocol. However, in a recent study, we applied inflations to 35 cmH_2_O twice a minute for an hour to test the effects of extended mechanical forces on lung structure and function (Szabari et al., 2012). Since the differences between lung structure and function with and without such inflations were smaller than the difference between those at 2 and 7 days after treatment, we are confident that the brief inflations to 25 cmH_2_O would not noticeably affect the lung. There are also limitations to our study. First, although the mouse is the most often used species in emphysema research, the structure of the lung and the response of the immune system to stimuli are different from those in humans. For example, we clearly see a difference in inflammatory cell distribution and activation in our study (Figure 8 and Table 4) whereas no clear regional differences were found in human surgical pneumonectomy specimens (Wright, 1988). Due to the small size of the mouse lung, any effect of gravity on tissue deterioration is expected to be much smaller than in the human lung. The elastase treatment produces a rapid development of emphysema and hence it does not mimic the slowly progressing effects of cigarette smoke. Consequently, the pattern of lung tissue destruction and remodeling is also different in the two mouse models of emphysema (Lopes et al., 2013). Characterizing structure from 2-dimensional images has certain disadvantages because it overestimates true airspace variability (Parameswaran et al., 2009). Additionally, while stereologic methods have been used to characterize the emphysematous lung structure (Ochs, 2014), we did not use such an approach. The reason is that the mechanism behind the progressive nature of emphysema is closely related to mechanical failure-induced structural heterogeneity (Winkler and Suki, 2011), which we have shown is best captured by the area weighted equivalent diameter, D_2_, (Parameswaran et al., 2006). Furthermore, since D_2_ is highly sensitive to heterogeneity, it is able to differentiate emphysema even in its very early stage and even without the necessity of knowing absolute lung volume. Nevertheless, in order to maintain consistency with the principles of stereology, we have used seven macroscopic regions and three to five randomly selected microscopic regions in our analysis.

We conclude that during the development of emphysema, microscale heterogeneity increases with the progression of the disease and gradually plays a dominant role in lung function. While the spatial distribution of the inflammatory response does not seem to follow that of the elastase deposition, it correlates with the strongest regional determinants of lung function. Hence inflammation appears to maintain processes that eventually lead to mechanical failure which in turn increases microscale heterogeneity. These results may help interpret lung function decline in terms of structural deterioration in human patients with emphysema.

### Conflict of interest statement

The authors declare that the research was conducted in the absence of any commercial or financial relationships that could be construed as a potential conflict of interest.
