# Supplementary material for: Scale dependence of structure-function relationship in the emphysematous mouse lung
Source: Front Physiol. 2015 May 12;6:146. doi: 10.3389/fphys.2015.00146 (PMC4428081; doi:10.3389/fphys.2015.00146)
Supplement: Supplementary file 1 [file Presentation1.PPTX]

## Slide 1
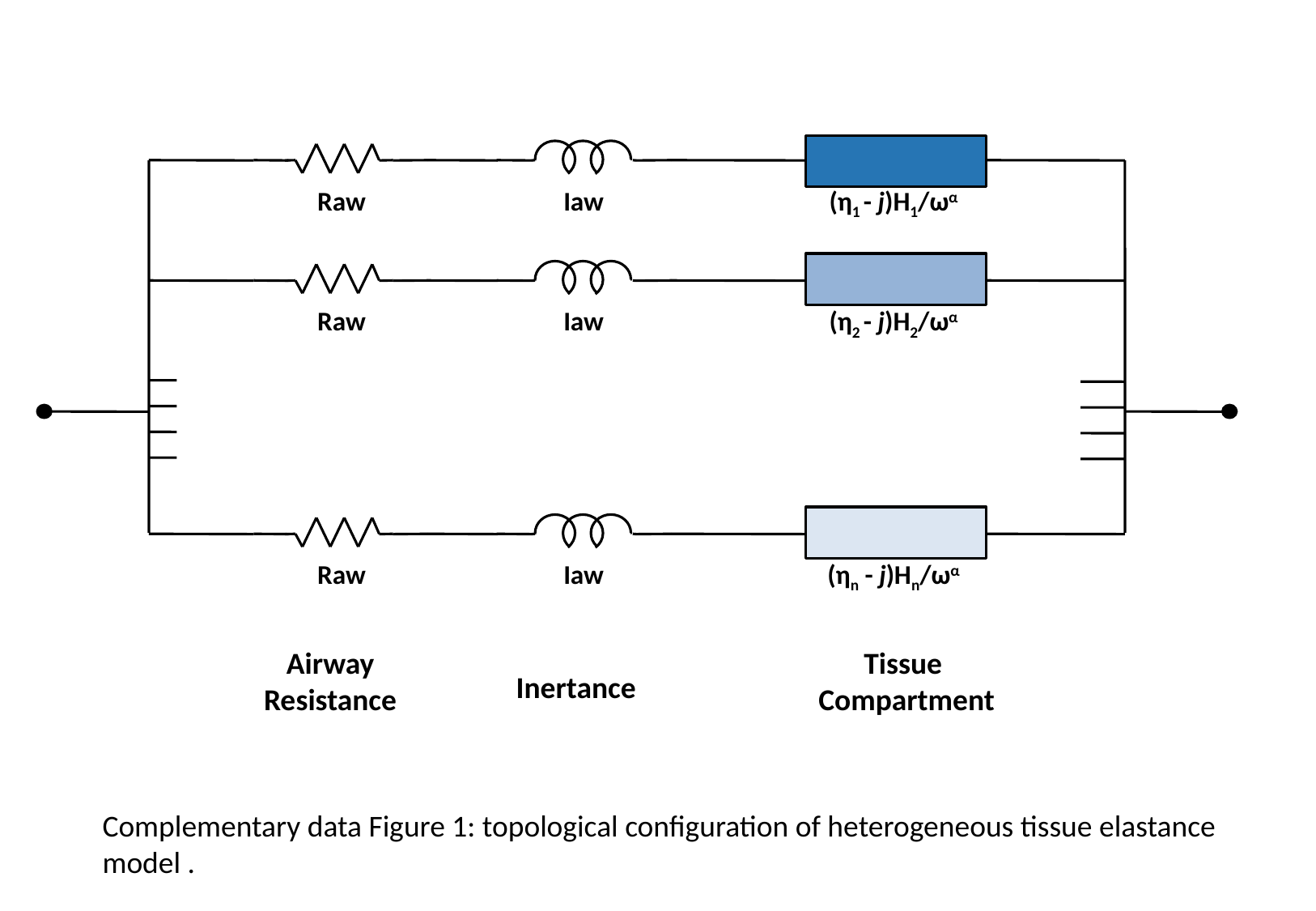

Raw
Iaw
(η1 - j)Η1/ωα
Raw
Iaw
(η2 - j)Η2/ωα
Raw
Iaw
(ηn - j)Ηn/ωα
Airway
Resistance
Tissue
Compartment
Inertance
Complementary data Figure 1: topological configuration of heterogeneous tissue elastance model .

## Slide 2
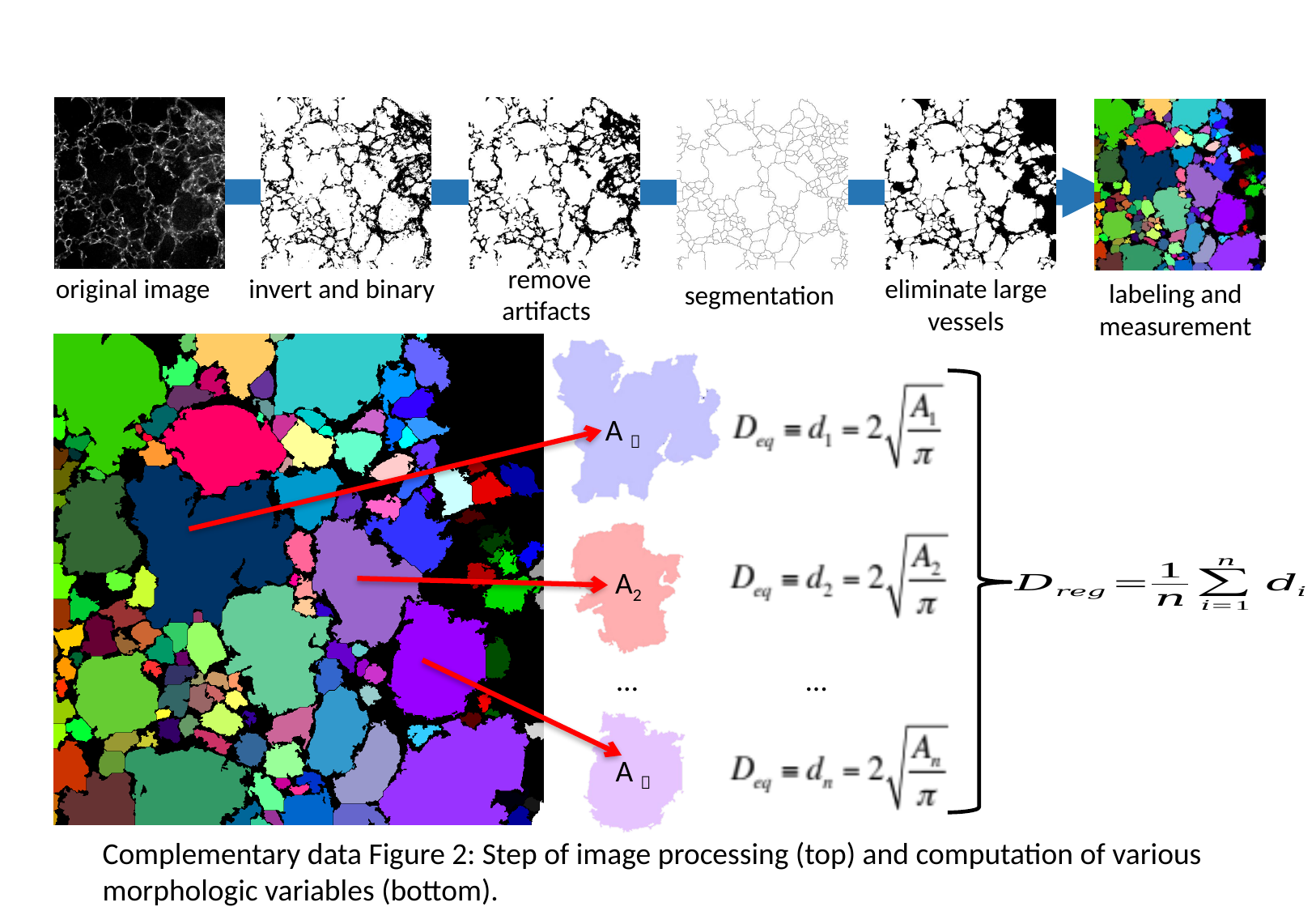

remove artifacts
eliminate large vessels
original image
invert and binary
labeling and measurement
segmentation
A１
A2
…
…
Aｎ
Complementary data Figure 2: Step of image processing (top) and computation of various morphologic variables (bottom).
